# Carotenoid Production by *Rhodotorula mucilaginosa* in Batch and Fed-Batch Fermentation Using Agroindustrial Byproducts

**DOI:** 10.17113/ftb.57.03.19.6068

**Published:** 2019-09

**Authors:** Tábita Veiga Dias Rodrigues, Thalita D. Amore, Erika Carvalho Teixeira, Janaina Fernandes de Medeiros Burkert

**Affiliations:** School of Chemistry and Food, Federal University of Rio Grande, 96203-900 Rio Grande, Brazil

**Keywords:** β-carotene, feeding strategies, corn steep liquor, sugar cane molasses, yeast

## Abstract

Carotenoids are natural pigments that can be produced through biotechnological processes. However, the costs are relatively high and can be minimized by using lower-cost substrates as alternative nutrient sources. The fed-batch fermentation is one of the techniques used to obtain a high biomass concentration and/or maximum production. Thus, the aim of this work is to produce carotenoids in batch and fed-batch fermentation with the yeast *Rhodotorula mucilaginosa* CCT 7688 using agroindustrial byproducts in the culture medium. Carotenoid production was increased using experimental designs, which modified the concentration of the agroindustrial medium. In batch production the highest concentrations of total carotenoids (1248.5 μg/L) and biomass (7.9 g/L) were obtained in the medium containing 70 g/L sugar cane molasses and 3.4 g/L corn steep liquor at 25 °C and 180 rpm in 168 h, demonstrating an increase of 17% when compared to the standard yeast malt medium (1200 μg/L). In the fed-batch production, different feeding strategies were tested with 30 g/L sugar cane molasses and 6.5 g/L corn steep liquor, reaching a total carotenoid production of 3726 μg/L and biomass concentration of 16 g/L. Therefore, the strategy of the fed-batch process resulted in an increase in the carotenoid production of approx. 400% compared to that in the batch process (740.3 μg/L). Thus, the *R. mucilaginosa* strain has the potential to produce carotenoids in agroindustrial medium.

## INTRODUCTION

Carotenoids are natural pigments found abundantly in nature whose isolation and characterization have identified more than 600 molecules, allowing a great applicability of these compounds, resulting in an increase in their use in the food, cosmetics and pharmaceutical industries (*1*). These compounds are responsible for the intense colouring of fruits, vegetables, flowers, algae, bacteria and fungi (*2*), which varies from yellow to red, and are the most studied natural pigments (*3*). The carotenoid world market in 2017 was US$ 1.5 billion with a forecast of US$ 2.0 billion for 2022. β-Carotene is the most commonly consumed carotenoid, with a market worth of US$ 261 million in 2010 and an estimation of US$ 334 million in 2018 (*4*).

The carotenoids, which are marketed as food additives and supplements, are mainly obtained by synthetic methods (*3*). However, their production by natural processes has increased due to the market demand for healthier foods with health benefits, in addition to the concern of the consumers for the use of chemical additives in food (*5*). The antioxidant capacity (*6*), the provitamin A activity, and the reduction of the risk of developing degenerative (*7*) and cardiovascular diseases (*8*) are some of the beneficial health effects provided by carotenoids.

These compounds obtained in bioprocesses can be produced by a diversity of microorganisms, such as microalgae (*9*-*11*) bacteria (*12*-*14*), fungi (*15*, *16*) and yeasts (*17*-*19*). However, the production of carotenoids using biotechnological processes still has a high cost. Therefore, an alternative to minimize the cost of this process is the utilization of agroindustrial byproducts as sources of alternative nutrients (*20*). The genus *Rhodotorula* has been studied with the use of low-cost substrates, such as sugar cane molasses (*21*), raw glycerol (*22*) and coffee grounds (*23*), to produce carotenoids. Although several microorganisms can biosynthesize carotenoids, not all of them are of interest for industry. However, yeasts need a source of carbon and nitrogen for biosynthesis, which is relatively simple when compared to other microorganisms (*24*).

The yeast *Rhodotorula mucilaginosa* utilized in this study was isolated previously (*25*) and it demonstrated the ability to produce carotenoids in media containing agroindustrial byproducts, such as rice parboiling wastewater, raw glycerol, sugar cane molasses and corn steep liquor (*26*), with antioxidant activity. Another factor that may increase the production of carotenoids is the use of the fed-batch fermentation. This process controls the substrate concentration, timing its addition at moments indicated as favourable, minimizing the effects of inhibition of the microorganism by the substrate and possibly improving the production of the biocompound (*27*, *28*).

Few studies are found in the literature that evaluate the carotenoid production using agroindustrial byproducts as potential sources of nutrients in fed-batch process using the yeast *Rhodotorula mucilaginosa.* In this work we aim to maximize the carotenoid production by *Rhodotorula mucilaginosa* in shake flasks with the use of agroindustrial byproducts and study different strategies of feeding in a fed-batch process.

## MATERIALS AND METHODS

### Microorganism

The yeast *Rhodotorula mucilaginosa* CCT 7688 used in this study was previously isolated (*25*) from environmental samples obtained in the region of Escudo Sul-Rio Grandense, Rio Grande do Sul (Brazil), identified and deposited at the André Tosello Tropical Culture Collection.

### Agroindustrial byproducts

The agroindustrial byproducts used in this study were corn steep liquor from Corn Products (Paraná, Brazil), sugar cane molasses obtained from Melaços Brasileiros (São Paulo, Brazil) and raw glycerol from the synthesis of biodiesel from BS Bios Indústria e Comércio de Biodiesel Sul Brasil S/A (Rio Grande do Sul, Brazil). A partial characterization of raw glycerol and substrates for yeast malt (YM) medium was performed, and the mass fractions of carbon and nitrogen were determined using a CHNS/O analyzer (Perkin Elmer 2400, Rodgau, Germany). Carbon in the sugar cane molasses was determined with the total organic carbon and total nitrogen analyzer (TOC-VCSH model; Shimadzu, Tokyo, Japan), and corn steep liquor was previously characterized (*26*).

### Maintenance and reactivation of microorganisms

The microorganisms were maintained in YM agar slant tubes with (in g/L): yeast extract 3, malt extract 3, peptone 5, agar 20 (all from KASVI, São José do Pinhais, PR, Brazil), glucose 10 (Synth, São Paulo, Brazil) and KNO_3_ 0.2 (Synth) at 4 °C (*29*) for 3 months. For reactivation, the microorganisms were transferred to the same medium and incubated at 25 °C for 48 h. From the tubes containing the microorganisms in YM agar slants, 1 mL of cell suspension in 0.1% sterile peptone water were added to 9 mL of YM broth and incubated before inoculation under the same conditions described previously (*29*).

### Carotenoid production in shake flasks

The inoculum was prepared in 250-mL Erlenmeyer flasks with 90 mL YM broth (*29*) and 10 mL previously grown culture and incubated (incubator model TE-420; Tecnal, Piracicaba, Brazil) at 25 °C and 150 rpm for 48 h or for the time needed to reach 10^8^ cells/mL, counted using a Neubauer chamber (Laboroptik, Lancing, UK) (*30*). Carotenoid bioproduction in batch and fed-batch processes was conducted in 500-mL Erlenmeyer flasks with 225 mL agroindustrial culture medium at an initial pH=6.0 and 10% inoculum (cultivation started with 10^7^ cells/mL). The conditions of the process were 25 °C, 180 rpm for up to 216 h (*31*). Culture samples were collected every 24 h to determine the pH, biomass concentration, total reducing sugars and production of carotenoids.

### Selection of agroindustrial medium for the production of carotenoids

Preliminary experiments were conducted to select the agroindustrial medium. Two formulations of agroindustrial byproducts were studied for the carotenoid production: corn steep liquor (35.6 g/L) with raw glycerol (6.6 g/L) and corn steep liquor (36.5 g/L) with sugar cane molasses (6 g/L) according to Cipolatti (*26*), and compared with YM medium (Table 1).

**Table 1:** Partial characterization of substrates, C:N ratio, carotenoid production by *Rhodotorula mucilaginosa* and productivity in different culture media at 25 °C, initial pH=6.0 and 180 rpm

Substrate	*w*/%			*γ*(medium)/(g/L)	C:N ratio	*w*_SC_/(μg/g)	*γ*_VC_/(μg/L)	*γ*(biomass)/(g/L)	*r*_b_/(g/(L·h))	*r*_vc_/(μg/(L·h))
C	N	H
CSL*	17.83	3.80	2.41	CSL 36.5 +SCM 6.0	6.04	(96.4±9.8)^a^	(336±10)^a^	(4.5±1.1)^a^	(0.10±0.01)^a^	(14.0±0.4)^a^
SCM	31.47	0.04	-							
RG	31.50	<0.07	2.27	CSL 35.6 +RG 6.6	6.20	(78.7±7.2)^a^	(297±15)^b^	(4.2±0.4)^b^	(0.07±0.00)^b^	(6.2±0.7)^b^
Yeast extract	41.26	11.45	5.35							
Malt extract	42.11	1.41	5.42	YM	8.28	176.3±4.5	1200±75	7.1±0.3	0.080±0.004	15.1±2.1
Peptone	12.16	2.70	1.46							

CSL=corn steep liquor, SCM=sugar cane molasses, RG=raw glycerol, YM=yeast malt medium; *composition according to Cipolatti (*26*); *w*_SC_=specific carotenoid mass fraction, *γ*_VC_=volumetric carotenoid concentration, r_b_=biomass productivity and r_vc_=volumetric carotenoid productivity, expressed as mean value±standard deviation (*N*=3); different letters in the same column indicate a significant difference (p<0.05) according to Student’s *t*-test

### Experimental design for batch carotenoid production

To maximize the batch carotenoid production, a study of the composition of the agroindustrial medium formulated with corn steep liquor and sugar cane molasses was conducted using two central composite designs (Table 2). The evaluated responses were the maximum volumetric concentration of carotenoids (μg/L) throughout the process, the specific mass fraction of carotenoids (μg/g) and biomass concentration (g/L), all determined at the same time. The experiments were performed in triplicate, under the incubation conditions described previously to validate the maximum carotenoid production using agroindustrial substrates.

**Table 2:** Coded levels and real values (in parentheses) of the first and second central composite design (CCD 2^2^) used for batch carotenoid production on agroindustrial media

First central composite design											
Assay	X_1_	X_2_		R_1_	R_2_		*t*_c_/h	R_3_	R_4_		R_5_
1	-1 (10)	-1 (3.5)		2.6	479		168	177.9	0.0		27.5
2	+1 (50)	-1 (3.5)		8.7	1040		144	119.2	5.1		106.9
3	-1 (10)	+1 (9.5)		3.1	407		120	128.0	0.0		13.2
4	+1 (50)	+1 (9.5)		7.2	1077		168	149.3	3.5		45.7
5	0 (30)	0 (6.5)		7.2	80		144	110.8	1.6		42.0
6	0 (30)	0 (6.5)		5.8	74		168	127.3	1.3		42.0
7	0 (30)	0 (6.5)		5.1	670		144	130.4	1.76		42.0
Second central composite design											
Assay	X_1_	X_2_	R_1_	DR_1_	R_2_	*t*_c_/h	DR_2_	R_3_	DR_3_	R_4_	R_5_
1	-1 (50)	-1 (0.58)	4.88	8.20	623	168	9.31	127.71	7.17	4.86	370.14
2	+1 (70)	-1 (0.58)	6.74	0.89	909	144	8.14	134.98	1.57	15.66	435.75
3	-1 (50)	+1 (3.41)	7.17	0.98	1077	168	6.87	163.62	6.50	3.14	109.26
4	+1 (70)	+1 (3.41)	8.10	4.94	1404	168	3.49	173.39	0.35	12.69	143.65
5	0 (60)	0 (2)	7.28	5.36	1209	168	11.75	166.15	6.72	4.57	192.39
6	0 (60)	0 (2)	7.07	2.55	1104	168	3.35	156.22	0.74	3.67	192.39
7	0 (60)	0 (2)	6.98	1.29	1141	168	6.49	165.61	6.15	3.55	192.39

X_1_=*γ*(SCM)/g/L, X_2_=*γ*(CSL)/(g/L), R_1_=*γ*(biomass)/(g/L), R_2_=*γ*VC/(µg/L), R_3_=*w*SC/(µg/g), R_4_=γ(total reducing sugar)/(g/L) and R_5_=C:N ratio (for abbreviations see legend of Table 1); DR_1_=relative deviation of biomass concentration, DR_2_=relative deviation of volumetric carotenoid concentration and DR_3_=relative deviation of specific carotenoids; *t_c_*=time of cultivation needed for maximum volumetric production of carotenoids

### Fed-batch fermentation

Based on the results of the batch fermentation, two production media were defined to evaluate fed-batch process: the first contained 3.5 g/L corn steep liquor and 70 g/L sugar cane molasses with one-pulse feeding in 168 h. The second culture medium contained 6.5 g/L corn steep liquor and 30 g/L sugar cane molasses with different feeding pulses (using the same amount of each component in the pulse): run 1 (one pulse at 96 h), run 2 (one pulse at 48 h), run 3 (one pulse at 72 h), run 4 (one pulse at 48 and one at 96 h), run 5 (one pulse at 72 and one at 120 h), run 6 (one pulse at 96 and one at 144 h) and run 7 (one pulse at 96 and one at 168 h). The feeding strategies for the fed-batch process were based on the procedure described by Chang *et al.* (*32*).

### Extraction and determination of total carotenoids

The biomass was recovered using centrifugation (Cientec CT-5000R; Belo Horizonte, Brazil) at 3439×*g* for 10 min and dried for 48 h at 35 °C to extract the carotenoids (*33*). It was subsequently macerated with a mortar and pestle and standardized with 115 mesh (*26*). The samples were frozen for 48 h at -18 °C (*33*). The cells were disrupted with dimethylsulfoxide (DMSO; Synth) as described by Michelon *et al.* (*30*). In tubes containing 0.05 g of biomass, 2 mL of DMSO were added at 55 °C and homogenized for 1 min in a vortex (Biomixer QL-901, Ningbo, PR China) at 15 min intervals for a total of 1 h. After cell disruption, 6 mL of acetone (Neon, Suzano, SP, Brazil) were added to stimulate the extraction and the suspension was centrifuged at 1745×*g* (CT-5000R; Cientec) for 10 min. The supernatant was separated, and the procedure was repeated until the cells were totally bleached. A volume of 10 mL of 20% NaCl solution (*m*/*V*) (Synth) and 10 mL of petroleum ether (Neon) were added to the supernatants. After the formation of two phases, the apolar phase was filtered with Na_2_SO_4_ (Neon) to form the carotenogenic extracts (*30*).

The total carotenoid mass fraction in the extracts was determined at 448 nm using spectrophotometer model SP-220 (Biospectro, Zhejiang, PR China) and expressed as its major component (β-carotene in petroleum ether with a specific absorbance of =2592 using the following equation (*34*):


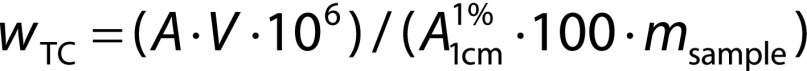


where *w*_TC_ is the mass fraction total of carotenoids (μg/g), *A* is the absorbance, *V* is the volume of carotenoids (mL), *m*_sample_ is the dried cell mass (g), and is the specific absorbance. The volumetric concentration of carotenoids (μg/L) was calculated using the mass fraction of total carotenoids (μg/g) multiplied by the biomass concentration (g/L).

### Determination of pH and biomass concentration

Aliquots were taken from the fermentation and centrifuged (1745×*g* for 10 min), the supernatants were separated for pH determination with a potentiometer (Quimis Q400MT; São Paulo, Brazil) as described by the AOAC official method 972.44 (*35*). The biomass concentration throughout the process was estimated by measuring the absorbance at 620 nm (SP-220; Biospectro) using a previously constructed standard biomass curve (g/L) (*36*).

### Determination of total reducing sugars

Total reducing sugars were determined spectrophotometrically at 540 nm (SP-220; Biospectro) with 3,5-dinitrosalicylic acid (DNS; Vetec, São Paulo, Brazil) as described by Miller (*37*) using a standard glucose curve. The sugar determination was performed in the culture medium previously centrifuged (CT 5000 R; Cientec) at 3439×*g* for 10 min. The agroindustrial medium containing sugar cane molasses was previously subjected to hydrolysis with 2 mL of 2 mol/L HCl (Neon) in boiling water (100 °C for 10 min), followed by the addition of 2 mL of 2 mol/L NaOH (Neon) to neutralize the acid (*38*).

### Determination of the kinetic parameters

The volumetric carotenoid productivity, *r*_vc_ (μg/(L·h)), and biomass productivity, *r*_b_ (g/(L·h)), were calculated using the following equations:

*r*_vc_=(*γ*_max_–*γ*_0_)/*t*_f_ /2/

and

*r*_b_=(X_max_–X_0_)/*t*_f_ /3/

where *γ*_max_ and *γ*_0_ are the maximum and initial volumetric concentrations of carotenoids (μg/L) respectively, *t*_f_ is fermentation time (h) at which the maximum volumetric concentration of carotenoids was obtained, X_max_ is biomass concentration (g/L) at *t*_f_, and X_0_ is initial biomass concentration (g/L).

### Statistical analysis

Data were analysed using Statistica software v. 5.0 (*18*). All analyses used a 95% confidence level (p<0.05). An analysis of variance (ANOVA) was used to estimate the statistical parameters. The mean value was compared by Tukey’s test at a 5% significance level. For the comparison between two treatments, the Student’s *t*-test (p<0.05) was used. Contour surfaces were drawn as described by Box *et al.* (*39*) and Rodrigues and Iemma (*40*).

## RESULTS AND DISCUSSION

### Characterization of substrates of the culture medium and influence on the carotenoid production

The partial characterization, including carbon and nitrogen mass fractions, of the substrates used in the formulations of the culture medium to produce carotenoids (Table 1) is consistent with the literature findings for these agroindustrial residues (*25*, *26*, *41*). The production of various biomolecules, such as lipids, rhamnolipids (*42*), exopolysaccharides (*43*) and carotenoids (*44*), can be influenced by the C:N ratio. The YM, corn steep liquor with raw glycerol and corn steep liquor with sugar cane molasses had a C:N ratio of 8.28, 6.20 and 6.04, respectively (Table 1). A C:N ratio higher than 5.0 may positively influence carotenoid production as described for *Phaffia rhodozyma* (*17*, *31*, *44*). Therefore, agroindustrial media with this C:N ratio are promising sources of alternative substrates for carotene bioproduction.

The highest specific mass fraction and volumetric concentration of carotenoids, as well as volumetric carotenoid productivity, were in the YM medium (Table 1), having a C:N ratio higher than that of the two agroindustrial media tested, which may have influenced the production of the carotenoids and biomass. However, carotenoid synthesis was similar in both agroindustrial media, with a C:N ratio close to 6.0 (Table 1), since there was no significant difference (p>0.05) in the specific carotenoid production. The corn steep liquor with sugar cane molasses was selected to maximize the carotenoid production because it resulted in a volumetric concentration, biomass concentration, biomass productivity and volumetric productivity higher by 12.0, 6.7, 55.7 and 30.0%, respectively, than of the corn steep liquor with raw glycerol.

### Maximization of carotenoid production

The use of an experimental design enables the study of the influence of the levels of one factor on the response variable. Thus, the primary effects of such a design may be simply calculated as the difference between the average value of the measurements made at the high level (+1) of the variable and the average value of measurements at the low level (-1) (*40*). The first experimental design was conducted using a central composite design (CCD) to evaluate the primary effects of the concentrations of sugar cane molasses and corn steep liquor in the medium on the production of carotenoids. The real and coded values of the investigated variables with the respective responses are shown in Table 2.

In the first central composite design the volumetric concentration of carotenoids ranged from 407 μg/L (assay 3) to 1077 μg/L (assay 4), the specific carotenoid mass fraction from 110.8 μg/g (assay 5) to 177.9 μg/g (assay 1), biomass concentration from 2.6 g/L (assay 1) to 8.7 g/L (assay 2), and the C:N ratio of the production medium from 13.2 (assay 3) to 106.9 (assay 2). The final concentration of the total reducing sugars in assays 1 and 3 was not detected, indicating the total sugar consumption by the yeast, while it was 5.1 g/L in assay 2 (initial higher concentration of sugar cane molasses).

**Fig. 1:**
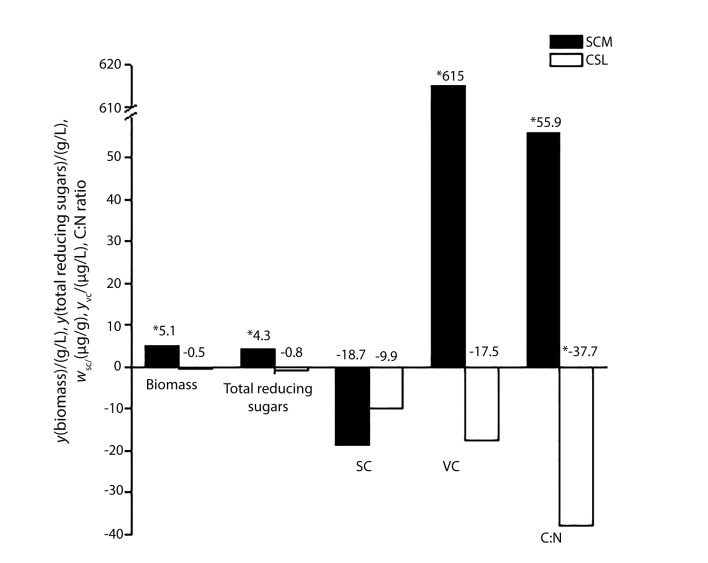
Effects of the variables of sugar cane molasses (SCM) and corn steep liquor (CSL) on the responses of volumetric carotenoid (VC) concentration, specific carotenoid (SC) mass fraction, biomass concentration, total reducing sugars and C:N ratio in the first central composite design (CCD). *significantly different (p<0.05)

The analysis of the primary effects (Fig. 1) indicated that the increase in sugar cane molasses concentration (from 10 to 50 g/L) positively influenced (p<0.05) the production of carotenoids (615 μg/L), biomass concentration (5.1 g/L), total reducing sugar concentration in the culture (4.3 g/L) and the initial C:N ratio (60) in the production medium. A different behaviour was observed with the increase of corn steep liquor concentration (from 3.5 to 9.5 g/L), which did not significantly influence response variables (p>0.05), except the C:N ratio, which decreased to approx. 40. Variations in the concentrations of sugar cane molasses and corn steep liquor in the studied range did not influence the carotenoid synthesis significantly (p>0.05), which was confirmed by specific carotenoid mass fractions. The maximum of biomass and volumetric carotenoid concentrations was achieved in assays 2 (8.7 g/L and 1040 μg/L respectively) and 4 (7.2 g/L and 1077 μg/L respectively) (Table 2). In general, the initial C:N ratio in the production medium above 40 increased the cell growth and volumetric concentration of carotenoids.

The concentration of sugar cane molasses probably had a significant effect on the evaluated responses because its composition is considerably richer in carbohydrates than of corn steep liquor (Table 1). Carbon source is one of the most frequently studied variables because it influences the production of carotenoids, by affecting acetyl coenzyme A (CoA) synthesis, which converts to mevalonic acid, the first precursor of carotenoid production (*20*).

Thus, for the maximization of carotenoid production, a second CCD was conducted, where the concentration range of the sugar cane molasses was increased and that of the corn steep liquor decreased (Table 2). This experimental design demonstrated a variation in the responses, since the volumetric concentration of carotenoids varied from 623 µg/L (assay 1) to 1404 µg/L (assay 4), mass fraction of specific carotenoids from 127.71 µg/g (assay 1) to 173.39 µg/g (assay 4), biomass concentration from 4.88 g/L (assay 1) to 8.10 g/L (assay 4), C:N ratio from 109.26 (assay 3) to 435.75 (assay 2) and the final concentration of the total reducing sugars from 3.14 g/L (assay 3) to 15.66 g/L (assay 2).

The C:N ratio plays an important role in the synthesis of secondary metabolites (*45*). However, a lower concentration of nitrogen allows a higher C:N ratio in the culture medium, which can affect the reduction in cell growth and the production of carotenoids (*31*, *44*). The higher C:N ratio (370.14 and 435.75 in assays 1 and 2 in the second CCD; Table 2) negatively influenced cell growth and carotenoid production. Similar results were obtained by Saenge *et al.* (*46*), who studied different C:N ratios (140, 160 and 180) and found that the C:N ratio of 180 allowed higher carotenoid production, close to that obtained in this study (ratios 110-190 in assays 3 to 7 in the second CCD, Table 2).

A model fitting was accomplished in the second CCD (Table S1) with the independent variables (corn steep liquor and sugar cane molasses concentrations) and responses (biomass concentration, specific carotenoid mass fraction and volumetric carotenoid concentration).

On the basis of the ANOVA (Table S1), Eqs. 4, 5 and 6 were established resulting in first-order models to describe the volumetric carotenoid concentration in µg/L, specific carotenoid mass fraction in µg/g and biomass concentration in g/L, respectively, as a function of corn steep liquor and sugar cane molasses concentrations:

*γ*_vc_=1067.7+151.50·X_1_+235.50·X_2_ /4/

*w*_SC_=154.85+18.75·X_2_ /5/

*γ*_biomass_=6.38+0.7·X_1_+0.91·X_2_ /6/

where X_1_ is sugar cane molasses concentration in g/L, and X_2_ is corn steep liquor concentration in g/L.

**Fig. 2:**
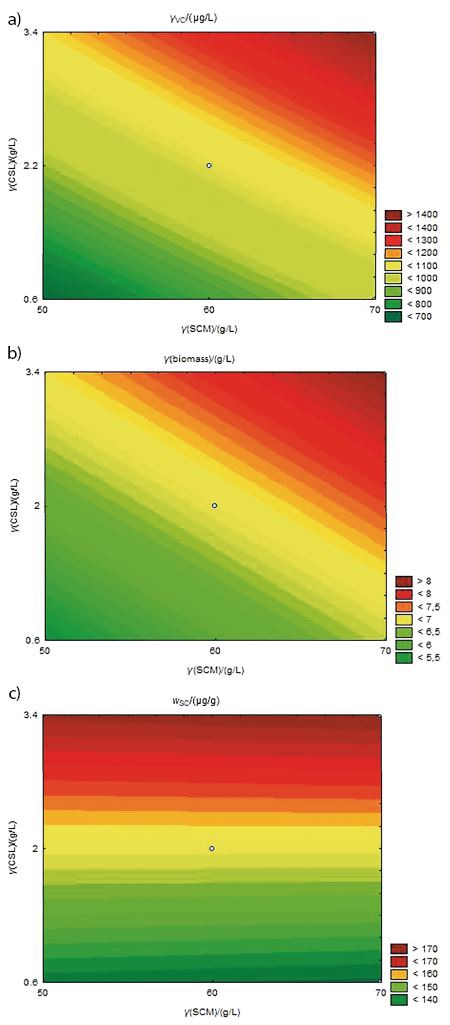
Contour curves of: a) volumetric carotenoid concentration, b) biomass concentration obtained by CCD and c) specific carotenoid concentration as a function of the corn steep liquor (CSL) and sugar cane molasses (SCM) concentrations

The pure error was low, indicating good reproducibility of the experimental data. Based on the F-test, the models are predictive, since the calculated F-value is higher than the critical F-value (3.4-, 2.48- and 2.91-fold for volumetric carotenoid, specific carotenoid mass fraction and biomass concentration, respectively), and the regression coefficients (0.93, 0.87 and 0.96 for volumetric carotenoid concentration, specific carotenoid mass fraction and biomass concentration, respectively) are considered satisfactory (*47*). Coded models were used to generate contour curves (Fig. 2).

The maximization of the volumetric concentration of carotenoids (Fig. 2a) and biomass concentration (Fig. 2b) occurred with the increase in the concentration of corn steep liquor and sugar cane molasses. The maximum specific carotenoid mass fraction (Fig. 2c) was independent from the concentration range of the sugar cane molasses, but higher in concentrations of corn steep liquor.

These results are observable in assay 4 (second CCD, Table 2), which used the maximum levels (level+1) of the studied variables, verifying the highest volumetric carotenoid concentration of 1404 µg/L, specific carotenoid mass fraction of 173.39 µg/g, and biomass concentration of 8.1 g/L.

### Validation of the model for the carotenoid bioproduction

**Fig. 3:**
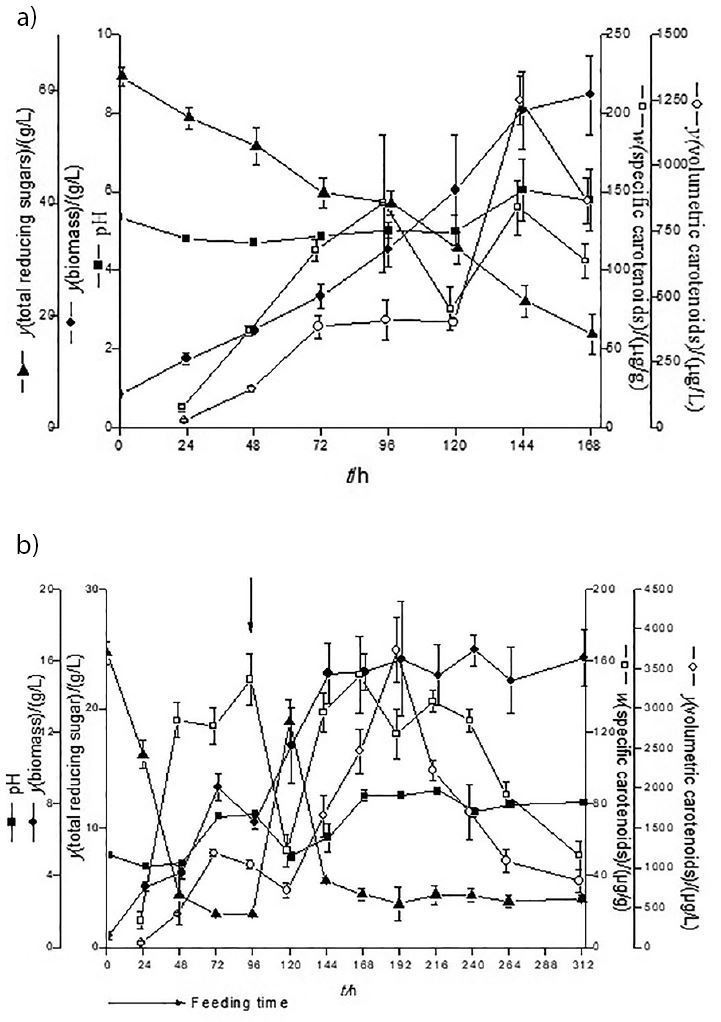
Kinetics of the carotenoid production by *Rhodotorula mucilaginosa* at 25 °C, 180 rpm and an initial pH=6.0: a) *γ*(sugar cane molasses)=70 g/L and *γ*(corn steep liquor)=3.4 g/L used to validate the empirical models (*N*=3), b) *γ*(sugar cane molasses)=30 g/L and *γ*(corn steep liquor)=6.5 g/L in a fed-batch fermentation at feeding time *t*=96 h

Fig. 3 shows the kinetics of the carotenoid production by *Rhodotorula mucilaginosa*. To find the highest volumetric concentration and specific mass fraction of carotenoids and biomass concentration, models generated by Eqs. 4, 5 and 6 were validated with the following composition of the production medium: 70 g/L sugar cane molasses and 3.4 g/L corn steep liquor (Fig. 3a). These assays demonstrated the expected behaviour in carotenoid bioproduction. The initial pH showed a small decrease in the first 24 h, reaching at the end an average of 6.0. Culture medium had an initial C:N ratio of 143.65, and the total reducing sugars decreased by an average of 50% in the first 96 h, not being fully consumed at the end of the process (15 g/L). A maximum biomass concentration of 7.9 g/L was achieved, approx. 2.5% less than the model (Eq. 6) had predicted (8.2 g/L). The volumetric carotenoid concentration reached a maximum of 1248.5 μg/L in 144 h, 14.2% less than predicted by Eq. 4 (1454.7 μg/L). Under the same conditions, the specific carotenoid mass fraction was 152.5 μg/g, 13.8% less than predicted by Eq. 5 (173.6 μg/g).

The relative deviations obtained during the experimental validation were less than 20% from those predicted by the models. Therefore, the experimental results fit well with the proposed models (*40*). The models generated for the other responses in this study were not predictive.

The agroindustrial medium used by *R. mucilaginosa* in this study was 70 g/L sugar cane molasses and 3.4 g/L corn steep liquor, with a C:N ratio of approx. 140, reaching a maximum of total carotenoid production 1248.5 μg/L (152.5 μg/g) and biomass concentration 7.9 g/L, at 25 °C and 180 rpm with initial pH=6.0 in 144 h. Under these conditions similar results to those on standard YM medium (1200 μg/L, Table 1) were achieved, using only two agroindustrial byproducts. In addition, a 270% increase in the production of the volumetric carotenoid concentration and 75% in the biomass production was achieved compared to the initial agroindustrial medium with initial C:N ratio 6.0 (36.5 g/L corn steep liquor with 6.0 g/L sugar cane molasses, Table 1).

The increase in the volumetric concentration of carotenoids achieved in this study through experimental design was at least 3 and 5 times higher than that reached by Otero (*25*) and Cipolatti (*26*), respectively, with the same yeast strain and similar agroindustrial substrates in other concentrations.

### Carotenoid production in the fed-batch fermentation

Two culture media were selected for the study of the fed-batch fermentation. One was the previously optimized medium (3.4 g/L corn steep liquor with 70 g/L sugar cane molasses), and the second culture medium (6.5 g/L corn steep liquor with 30 g/L sugar cane molasses) in batch process reached approx. 60% of the optimum carotenoid production (run 5 to run 7 in the first central composite design in Table 2). Additionally, in this process a depletion of total reducing sugars occurred in 96 h (data not shown), making it interesting for fed-batch production with the pulse feeding with 25 mL of medium (*32*). The biomass concentration increased approx. by 70% (run 1 to 2) and 168% (run 3 to 4) in the fed-batch batch compared to the batch process (Table 3) in both media.

**Table 3:** Biomass concentration and carotenoid production on agroindustrial media in batch and fed-batch fermentation with different feeding strategies

Trial	*γ*(medium)/(g/L)	Process	Feeding pulse	*γ(*biomass)/(g/L)	*w*_SC_/(µg/g)	*γ*_VC_/(µg/L)	*t*_c_/h
1	CSL 3.4+SCM 70	Batch	-	(7.9±0.9)^b^	(152±13)^a^	(1248± 94)^c^	144
2		Fed-batch *t*=168 h	50	(13.7±1.2)^a^	(139±22)^a^	(2229±1534)^b^	192
3	CSL 6.5+SCM 30	Batch	-	(6.0±0.8)^b^	(123±9)^a^	(740±55)^d^	144
4		Fed-batch *t*=96 h	25	(16.1±4.5)^a^	(118.8±13.7)^a^	(3726.7±506.0)^a^	192

CSL=corn steep liquor, SCM=sugar cane molasses; *w*_SC_=specific mass fraction of carotenoids, *γ*_VC_=volumetric concentration of carotenoids, *t*_c_=time of cultivation; different letters in the same column indicate a significant difference (p<0.05) according to Tukey’s test

During the follow-up of the carotenoid production in fed-batch fermentation with feeding in 96 h (Fig. 3b), the pH decreased during the first 24 h of culture after a gradual increase. With feeding, the pH dropped again in the first hours and gradually increased throughout the process. This pH change was also observed by Cipolatti (*26*). The decline in the pH probably occurs as a consequence of cell development and the release of compounds, such as acetic acid, alcohol or citric acid cycle intermediates during the adaptation phase (*24*).

**Fig. 4:**
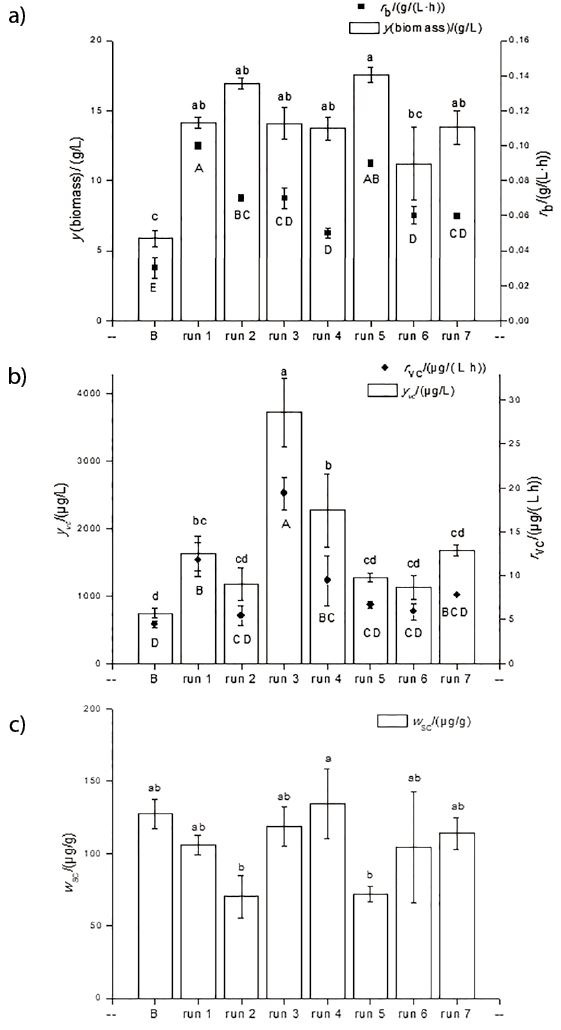
Comparison of: a) biomass concentration in batch and fed-batch fermentations with 6.5 g/L corn steep liquor and 30 g/L sugar cane molasses medium, b) volumetric concentration of carotenoids and c) specific carotenoid mass fraction. B=batch process, fed–batch run (*t*/h): 1) 48, 2) 72, 3) 96, 4) 48 and 96, 5) 72 and 120, 6) 96 and 144, 7) 96 and 168. The results are expressed as mean values±standard deviations (*N*=3); different letters (lowercase for biomass and volumetric carotenoid concentrations and uppercase for biomass and volumetric carotenoid productivity) indicate a significant difference (p<0.05) according to Tukey’s test

The synthesis of the carotenoids was not significantly influenced (p>0.05) by the fed-batch process or the composition of the culture medium, since there was no variation in the mass fraction of specific carotenoids. Similar was observed for the volumetric carotenoid concentration, where a significant increase (p<0.05) was observed during fed-batch production by 78% (trials 1 to 2 in Table 3) and 400% (trials 3 to 4 in Table 3). Therefore, the production medium containing 6.5 g/L corn steep liquor with 30 g/L sugar cane molasses (run 4 in Table 3) was the best for carotenoid and biomass production in the fed-batch process with different feeding strategies (Fig. 4).

There was an increase (p>0.05) of about 150% in biomass accumulation in all feeding strategies on medium containing 6.5 g/L corn steep liquor with 30 g/L sugar cane molasses, when compared to that of the batch fermentation (Fig. 4a). However, it was observed that there was no significant difference (p<0.05) in the carotenoid synthesis between the evaluated strategies (Fig. 4c). The highest volumetric carotenoid concentration was in run 3 with the feeding pulse in 96 h of culture (3726.7 μg/L), when a significant increase (p<0.05) compared to the other feeding strategies and from the batch process was observed (Fig. 4b). In relation to the YM standard medium (Table 1), there was a 3-fold increase in carotenoid production (1200 μg/L). The volumetric productivity reached 19.40 μg/(L·h) in run 3, which was significantly (p<0.05) higher than under other conditions. The biomass productivity varied from 0.05 to 0.1 g/(L·h), depending on feeding strategy. Colet *et al.* (*48*) obtained biomass productivity between 0.05 and 0.085 g/(L·h), which was similar to the results of this study.

Colet *et al.* (*48*) evaluated the production of carotenoids in fed-batch fermentation by *Sporidiobolus salmonicolor* in a bioreactor with 1 L working volume. The composition of the medium was: peptone 15 g/L, malt extract 5 g/L and glycerol 80 g/L. The maximum total carotenoid concentration obtained in their studies was 4400 μg/L in 96 h with a 112.5 mL feed every 12 h (Table 4 (*18*, *48*-*50*)). Dias *et al.* (*51*) studied the production of lipids and carotenoids by *Rhodosporidium toruloides* in a batch and fed-batch fermentation in a bench bioreactor with 5 L working volume. The maximum production obtained was 33.4 mg/L under conditions of initial pH=5, temperature 30 °C with strategy of one pulse feeding at the end of the batch culture in the medium containing 9 g/L MgSO_4_·7H_2_O, 20 g/L yeast extract and 60 g/L glucose.

**Table 4:** Carotenoid production obtained by cultivation of red yeasts on different substrates and operating conditions

Strain	Substrate	Operating conditions	Cultivation method	*γ*_VC_/(μg/L)	Reference
*Rhodotorula mucilaginosa*	Sugar cane molasses	Incubated in a shake flask at 25 °C, 180 rpm and initial pH=6.0	Batch	1248.5	This work
*Rhodotorula mucilaginosa*	Sugar cane molasses	Incubated in a shake flask at 25 °C, 180 rpm and initial pH=6.0	Fed-batch	3726.0	This work
*Sporidiobolus salmonicolor*	Crude glycerol, maceration water and rice parboiling water	Stirred tank bioreactor at 25 °C, initial pH=4.0 and 180 rpm	Batch	7388.0	(*49*)
*Rhodotorula glutinis*	Wastewater food industry	Incubated in a shake flask at 25 °C,115 rpm and initial pH=5.5	Batch	1200.0	(*50*)
*Sporidiobolus pararoseus*	Sugar cane molasses	Stirred tank bioreactor at 25 °C, initial pH=6.0, and 158 rpm	Batch	1969.3	(*18*)
*Sporidiobolus pararoseus*	Sugar cane molasses	Incubated in a shake flask at 27.5 °C, 150 rpm and initial pH=4.0	Batch	565.0	(*18*)
*Sporidiobolus salmonicolor*	Glycerol	Stirred tank bioreactor at 25 °C, 180 rpm and initial pH=4.0	Fed-batch	4400.0	(*48*)

Table 4 summarizes the carotenoid production findings in the literature and compares them with the results obtained in this work. The study demonstrated promising results considering that the medium contains only two agroindustrial byproducts, sugar cane molasses and corn steep liquor, and was conducted in shake flasks.

## CONCLUSIONS

In this study, it was possible to use agroindustrial byproducts (sugar cane molasses, corn steep liquor and raw glycerol) to produce carotenoids by *Rhodotorula mucilaginosa*. The carotenoid production in the batch fermentation in the medium containing 70 g/L sugar cane molasses and 3.4 g/L corn steep liquor reached a maximum of 1248.5 μg/L (152.5 μg/g), with 7.9 g/L biomass concentration. The fed-batch fermentation using the agroindustrial culture medium (6.5 g/L corn steep liquor with 30 g/L sugar cane molasses) with a 96-hour feed gave promising results of 3726.7 μg/L carotenoids (118.8 μg/g). Therefore, this demonstrates the potential of using agroindustrial byproducts as an alternative source of nutrients in the batch and fed-batch fermentation. In summary, our study demonstrated the feasibility of minimizing costs of the production medium, adding value to these byproducts, and possibly decreasing the generation of waste from industrial processes and reducing their environmental impact.
